# ApoE Alzheimer’s Disease Aβ-amyloid plaque morphology varies according to APOE isotype

**DOI:** 10.21203/rs.3.rs-2524641/v1

**Published:** 2023-02-08

**Authors:** Ina Caesar, K Peter R Nilsson, Per Hammarstrom, Mikael Lindgren, Stefan Prokop, Frank L Heppner, James Schmeidler, Vahram Haroutunian, David M Holtzman, Patrick R Hof, Sam Gandy

**Affiliations:** Icahn School of Medicine at Mount Sinai; Linköping University: Linkopings universitet; Linköping University: Linkopings universitet; Norwegian University of Science and Technology: Norges teknisk-naturvitenskapelige universitet; University of Florida College of Medicine; Charite Universitatsmedizin Berlin Campus Charite Mitte: Charite Universitatsmedizin Berlin; Icahn School of Medicine at Mount Sinai; Icahn School of Medicine at Mount Sinai; Washington University In Saint Louis: Washington University in St Louis; Icahn School of Medicine at Mount Sinai; Icahn School of Medicine at Mount Sinai

**Keywords:** Alzheimer’s disease (AD), apolipoprotein E (APOE, gene; apoE, protein), hyperspectral fluorescence imaging, luminescent conjugated oligothiophene (LCO), plaque, Cerebral amyloid angiopathy (CAA), neurofibrillary tangle (NFT)

## Abstract

**Background:**

The apolipoprotein E (*APOE*, gene; apoE, protein) ε4 allele is the most common identified genetic risk factor for typical late-onset sporadic Alzheimer’s disease (AD). Each *APOE* ε4 allele roughly triples the relative risk for AD compared to that of the reference allele, *APOE* ε3.

**Methods:**

We have employed hyperspectral fluorescence imaging with an amyloidspecific, conformation-sensing probe, p-FTAA, to elucidate protein aggregate structure and morphology in fresh frozen prefrontal cortex samples from human postmortem AD brain tissue samples from patients homozygous for either *APOE* ε3 or *APOE* ε4.

**Results:**

As expected *APOE* ε4/ε4 tissues had significantly larger load of CAA than *APOE* ε3/ε3. *APOE* isoform-dependent morphological differences in amyloid plaques were also observed. Amyloid plaques in *APOE* ε3/ε3 tissue had small spherical cores and large corona while amyloid plaques in *APOE* ε4/ε4 tissues had large irregular and multilobulated plaques with relatively smaller corona. Despite the different morphologies of their cores, the p-FTAA stained *APOE* ε3/ε3 amyloid plaque cores had spectral properties identical to those of *APOE* ε4/ε4 plaque cores.

**Conclusions:**

These data support the hypothesis that one mechanism by which the *APOE* ε4 allele affects AD is by modulating the macrostructure of pathological protein deposits in brain. *APOE* ε4 is associated with a higher density of amyloid plaques (as compared to *APOE* ε3). We speculate that multilobulated *APOE ε4*-associated plaques arise from multiple initiation foci that coalesce as the plaques grow.

## Background

Alzheimer’s disease (AD) is characterized by accumulation of aggregated extracellular deposits of amyloid-β peptide (Aβ) [1, 2]. Assembly states for Aβ in the AD brain include classical fibrils containing highly structured β-pleated sheets and less structured oligomeric species [3, 4]. Current evidence indicates that different conformations of Aβ specify different degrees of neurotoxicity [3,4]. In humans with cerebral amyloidosis and in some transgenic mouse models, brain amyloid plaque density varies in an *APOE* ε4 dosedependent manner [5–8, 11]. Aβ clearance in the brain interstitial fluid is reduced in mice with *APOE* ε4 alleles [9, 10], but the molecular basis for this impairment in Aβ clearance is unknown. A direct protein-protein interaction between apoE and Aβ formed the basis for the biochemical observation that led to the genetic investigation in which AD risk was first linked to *APOE* isotype [11]. Along this same line, association of apoE isotype with Aβ oligomerization has been reported [12].

The recent development of novel molecular probes has enabled the spectral distinction of a wide range of different structures associated with deposits of amyloids of diverse amino acid sequences [13–20]. In the case of prion amyloid, compounds known as luminescent conjugated oligothiophenes (LCOs) provided the first imaging methodology for defining the prion protein conformers that underlie the prion strain phenomenon [13, 20, 21]. Upon binding, the LCOs intercalate within the amyloid deposits [16, 19, 21], and, based on the individual molecular structures of these deposits, the LCO adapts its flexible thiopheneconjugated backbone as a function of the molecular structure of the deposits. This adaptation is observed as a protein structure-dependent change in the fluorescence emitted by the dye. By hyperspectral imaging, the LCO emission profile can be analyzed and used to infer properties related to the conformation of the protein deposit [22]. LCO combinations have also been applied to reveal variations in Aβ-amyloid polymorphs in human AD brains from different familial subtypes [27] and in sporadic AD with different disease progression rates [28].

In this study, we have employed an anionic pentameric LCO, p-FTAA (4’,3”‘-bis-carboxymethyl-[2,2’;5’,2”;5”,2”‘;5”‘,2”“]quinquethiophene-5,5”“-dicarboxylic acid) [16], to investigate the differences in the molecular structure as well as the morphologies of amyloid plaques, neurofibrillary tangles (NFTs), and vascular amyloid (CAA) in the brains of postmortem AD tissue samples from patients homozygous for *APOE* ε3 or *APOE* ε4.

## Methods

### Cohort A.

Matched pairs of fresh frozen prefrontal cortices from the Charles F. and Joanne Knight Alzheimer’s Disease Research Center Tissue Resource, at Washington University School of Medicine (St. Louis, MO, USA) were used for this study. Brains were from patients who were homozygous for either *APOE* ε3 or *APOE* ε4, but otherwise matched for age, gender, Clinical Dementia Rating (CDR) scores, and disease duration (Table 1). Cryosections (20 μm) were cut and stained with pFTAA according to a standard protocol [17].

Cohort B.

Matched pairs of fresh frozen prefrontal cortex from the Alzheimer’s Disease Research Center Mental Illness and Alzheimer’s Disease Brain Bank, at the Icahn School of Medicine at Mount Sinai (New York, NY, USA) were used for this study. Brains were from patients who were homozygous for either *APOE* ε3 or *APOE* ε4, but otherwise matched for age, gender, and CDR scores (Table 2). Cryosections (20 μm) were cut and stained with pFTAA according to a standard protocol [17].

### Initial processing.

The postmortem AD tissue samples were matched for gender, severity, and duration of illness, and differed only by being homozygous either for the *APOE* ε4 allele (*APOE* ε4/ε4) or for the *APOE* ε3 allele (*APOE* ε3/ε3). A standard, discovery/validation biomarker strategy was employed. The discovery cohort (cohort A) was used for all extensive data analyses and to generate hypotheses, while cohort B was used to confirm in an independent population the observations made in the initial, discovery cohort.

### Statistical analyses.

Within each pair, the samples for *APOE* ε3 or *APOE* ε4 were compared with the two-tailed unpaired *t* test. The six matched samples within the same cohort were compared with the two-tailed paired *t* test of the respective subjects’ means. The two cohorts were compared with the two-tailed unpaired *t* test of six differences for each cohort to assess the differences between the paired subjects’ means. Statistical significance was set at α = 0.05 for the entire study.

### Amyloid load factor.

Semiquantitative amyloid load factors were generated for each sample. To estimate the frequency of the different forms of plaques, NFTs, and vascular amyloid within a particular sample, an amyloid load factor was defined as: undetectable (0), sparse amount (1), moderate amount (2), or frequent (3), for each type of lesion. Representative micrographs of amyloid load factors 1, 2, and 3 for plaques are shown in Figure 2b–d. The amyloid load factor is reported as a mean score with standard deviation (SD). The matched samples within the same cohort were compared with the two-tailed paired *t* test. The two cohorts were compared with two-tailed unpaired *t* test.

### Hyperspectral imaging of amyloid stained with p-FTAA.

A fluorescence microscope (Leica Microsystems, Bannockburn, IL, USA), with 405/40 nm, 480/20 nm, and 560/40 nm long-pass emission filters, attached with a spectra camera (Applied Spectra Imaging, Carlsbad, CA, USA) was used to collect spectral micrographs. The excitation wavelengths were chosen based on p-FTAA’s specific properties and hyperspectral micrographs with a cut-off at 45 nm after excitation and up to 700 nm were collected for all three excitations. The 405-nm excitation contributes to the secondary Aβ-specific emission peak at 511 nm as well as for the primary peaks for both Aβ (546 nm) and tau (550–560 nm) aggregates. The 480-nm excitation contributes to the primary peaks for both Aβ (546 nm) and tau (550–560 nm) aggregates, and the 560-nm excitation contributes to the tau-associated red shift of the emission spectra with a peak at 594 nm [18, 19].

All samples in cohort A were analyzed for five regions of interest of every type of neuropathological hallmark at all three excitations. In every micrograph, 9 individual spectra indicating individual structure were extracted. This gave a summary of 405 individual spectra for every sample, or 4,860 individual spectra for cohort A. Pair 1 in cohort B was analyzed for five regions of interest and pairs 2–6 in cohort B were analyzed for three regions of interest for every type of protein aggregate and for all three excitations. In every micrograph, 9 individual spectra indicating individual structures were extracted for all samples from cohort B. This accounts for a dataset of 405 individual spectra for the samples in pair 1 and 243 individual spectra for the samples in pairs 2–6, or 3,240 individual spectra for cohort B. The same exposure time was used for spectra collection of the same excitation. Spectral images under the noise level of (I_511_)^405^ = 200 were excluded from the dataset. To compensate for the stronger fluorescence signal emitted from the vascular amyloid compared to the fluorescence signal emitted from the plaques and NFTs, the intensity of the excitation lamp was decreased from 50% to 10%. Spectral micrographs for all three excitations, as well as additional hyperspectral micrographs of all the combined excitations combinations were computed using the SpectraView software (Applied Spectra Imaging, Carlsbad, CA, USA, 92008). The spectra were further analyzed with the GraphPad Prism 5.0d software (GraphPad Software, San Diego, CA, USA).

#### Emission fractions overall assessment:

The fluorescence intensity ratio of the emission peak at 546 nm (I_546_) and the emission peak at 594 nm (I_594_) for the triple excitation ${\left(\frac{{\text{I}}_{546}}{{\text{I}}_{594}}\right)}^{405:480:535}$ was used as an index of protein separation between amyloid of Aβ peptides and tau as previously reported [16–19, 24]. The emission fractions of the plaques, NFTs, and vascular amyloid were estimated as the mean divided by the standard error of the mean (SEM). Within each pair, the samples for *APOE* ε3 or *APOE* ε4 were compared with the two-tailed unpaired *t* test. The matched samples within the same cohort were compared with the two-tailed paired *t* test. The two cohorts were compared with the two-tailed unpaired *t* test.

#### Emission fractions optimized for amyloid β:

To analyze the structural differences of the plaque and vascular amyloid, the ratio of the optimal peaks of the fluorescence emission at 511 nm (I_511_) and at 546 nm (I_546_) for the 405 nm excitation ${\left(\frac{{\text{I}}_{511}}{{\text{I}}_{546}}\right)}^{405}$ was used.

#### Emission fractions optimized for NFTs:

To analyze the structural differences of NFTs, the ratio of the optimal peaks of the fluorescence emission at 550 nm (I_550_) and at 594 nm (I_594_) for the triple excitation ${\left(\frac{{\text{I}}_{550}}{{\text{I}}_{594}}\right)}^{405:480:560}$ was used. The emission fractions of samples within the same cohort were compared using two-tailed paired *t* test. The two cohorts were compared with two-tailed unpaired *t* test of the mean of the pairs. The variation of emission fractions was used as an indicator of structural heterogeneity and was analyzed as the coefficient of variation; $c_{v}=\frac{\sigma }{\mu }$, where σ is the SD and μ is the mean of the spectra shifts. The samples within the same cohort were compared with the two-tailed paired *t* test. The two cohorts were compared with the two-tailed unpaired *t* test.

## Results

**Figure 1.**
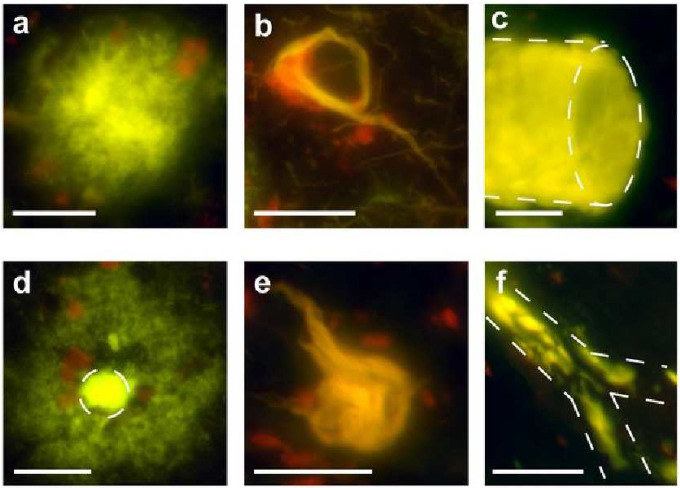
Hyperspectral emission micrographs of amyloid aggregates found in postmortem AD brain samples. Representative micrographs of (a) fibrillar plaque, (b) NFT, and (c) vascular amyloid in AD patients with *APOE* ε4/ε4 genotype. Representative micrographs of (d) dense-cored plaque, (e) NFT, and (f) vascular amyloid in AD patients with *APOE* ε3/ε3 genotype. Scale bars = 200 μm.

We classified amyloid plaques according to Dickson *et al* [23]. All samples homozygous for the *APOE* ε4/ε4 genotype had fibrillar plaques (Figure 1a) with large, irregular, multilobulated cores as the most abundant form of plaques. Occasionally, NFTs (apparently extracellular “ghost tangles”) were interwoven within the fibrillar plaques. NFTs were also abundant in all samples from *APOE* ε4/ε4 AD patients (Figure 1b). Vascular amyloid was present in all samples from subjects homozygous for *APOE* ε4, in both small (<100 μm in diameter) and large blood vessels (>500 μm in diameter) (Figure 1c), except in the *APOE* ε4/ε4 case of pair 1 in which no large blood vessels were found in the examined sections.

In all samples from patients with the *APOE* ε3/ε3 genotype, the dense-cored plaque with a halo-like corona was the most abundant form of plaque (Figure 1d). NFTs were present in five of the six pairs of samples from brains of patients homozygous for *APOE* ε3 (Figure 1e). Vascular amyloid was found in four of six samples from brains of patients homozygous for *APOE* ε3. Vascular amyloid in the brains of patients homozygous for *APOE* ε3 was preponderantly observed in very small blood vessels (<50 μm in diameter) and was especially frequent at the branch points of small blood vessels (<100 μm in diameter) (Figure 1f).

**Figure 2.**
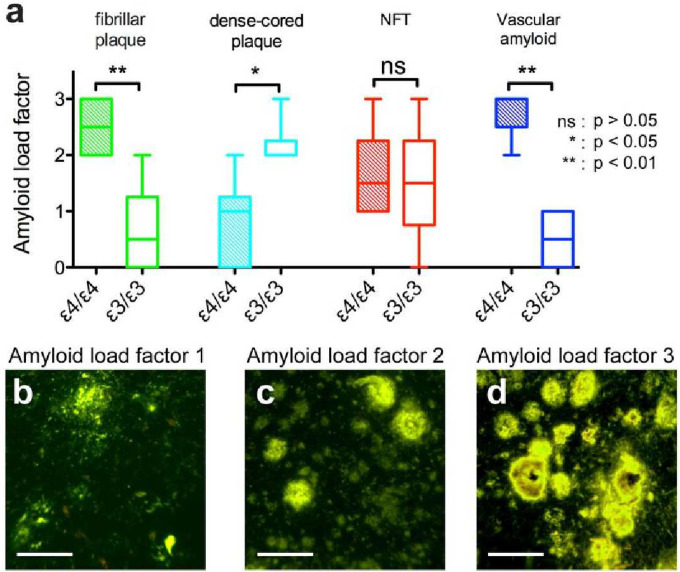
Amyloid load factor of amyloid deposits found in postmortem AD brain samples. (a) The amyloid load factor of plaques separated as fibrillar plaques (green), dense-cored plaques (cyan), NFT (red), and vascular amyloid (blue) of samples with *APOE* ε4/ε4 genotype (filled bars) and of samples with *APOE* ε3/ε3 genotype (open bars). The amyloid load factors represent undetectable (0), sparse densities (1), moderate densities (2), or heavy densities (3) of each type of lesion. Representative images of amyloid load factor 1, 2, and 3 of the plaques are showed in (b), (c), and (d) respectively.

To estimate the frequency of the different amyloid morphologies, an amyloid load factor was calculated. The amyloid load factor of the two different forms of plaques – namely the fibrillar, multi-lobular cored plaques (Figure 1a) and the dense-cored plaques (Figure 1d) – varied according to *APOE* isoform. The fibrillar plaques were more frequently found in the brains of patients homozygous for *APOE* ε4 (p < 0.001, two-tailed paired *t* test; green bars in Figure 2a), while the dense-cored plaques were more frequently found in brains of patients homozygous for *APOE* ε3 (p < 0.01, two-tailed paired *t* test; cyan bars in Figure 2a). Fibrillar plaques <250 μm in diameter were infrequently observed in *APOE* ε3/ε3 cases, while fibrillar plaques >500 μm in diameter were frequently observed in *APOE* ε4/ε4 cases. Vascular amyloid was observed in a much higher extent in the cases with the *APOE ε4/ε4* genotype (p < 0.001, two-tailed paired *t* test; blue bars in Figure 2a). Representative micrographs of amyloid load factor 1, 2, and 3 and of the range of plaque morphologies are shown in Figure 2b–d. Amyloid load factors in brains from cohort B are shown in Figure 4. There were no significant differences in the amyloid load factor distribution between cohort A and cohort B of the dense-cored plaques (p = 0.0761, two-tailed unpaired *t* test), the classic cored plaques (p = 0.4956, two-tailed unpaired *t* test), the NFTs (p = 0.8040, two-tailed unpaired *t* test), or for vascular amyloid (p = 0.1288, two-tailed unpaired *t* test). There was no significant difference in the density of NFTs as a function of *APOE* genotype (p = 0.7926, two-tailed paired *t* test; red bars in Figure 2a).

Based on earlier studies showing that the emission profile from p-FTAA can be utilized for hyperspectral separation of Aβ and tau deposits [16], the emission profiles from p-FTAA bound to the defined pathological entities were examined. Upon binding to Aβ aggregates, p-FTAA displayed a similar emission profile with two maxima at 511 nm and 546 nm as reported previously [16–19, 21]. In contrast, p-FTAA emission spectra from tau aggregates displayed red-shifted spectra relative to the emission spectra from Aβ with a pronounced shoulder at 594 nm [16–18]. Hence, emission fractions (ratios of the emitted light at these specific wavelengths) can be utilized for spectroscopic evaluation of Aβ and tau deposits. In addition, as the spectral separation of these pathological hallmarks can be enhanced by multiple wavelength excitations [18, 19], we utilized a set of filters (405/40 nm, 480/20 nm, and 560/40 nm long-pass) with distinct excitation wavelengths (405 nm, 480 nm, and 560 nm).

**Figure 3.**
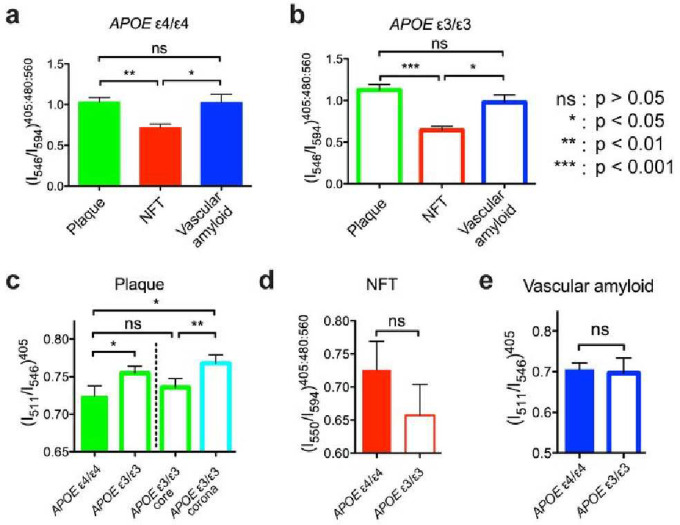
Spectral separation as a function of *APOE* genotype. (a) Spectral emission fraction of plaques (green), NFT (red), and vascular amyloid (blue) in AD patients with *APOE* ε4/ε4 genotype. (b) Spectral emission fraction of plaques (green), NFT (red), and vascular amyloid (blue) in AD patients with *APOE* ε3/ε3 genotype. Spectral separation using optimized emission fractions of (c) plaque, (d) NFT, and (e) vascular amyloid in AD patients with *APOE* genotype of ε4/ε4 (filled bars) or respective ε3/ε3 (open bars). The spectral separation of the dense-cored plaques from *APOE* ε3/ε3 AD patients are separated into a core (green) and a corona (cyan) fraction in (c). Error bars indicate SEM.

**Figure 4.**
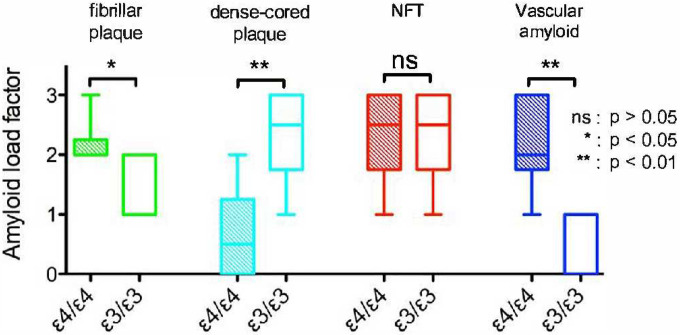
Amyloid load factor of amyloid aggregates found in postmortem AD brain samples of cohort B. The amyloid load factor of plaques separated as fibrillar plaques (green) and dense-cored plaques (cyan), NFT (red), and vascular amyloid (blue) of samples with *APOE* ε4/ε4 genotype (filled bars) and of samples with *APOE* ε3/ε3 genotype (open bars). The amyloid load factor is classified from samples as: undetectable (0), sparse amount (1), moderate amount (2), or frequent (3), for each type of lesion.

**Figure 5.**
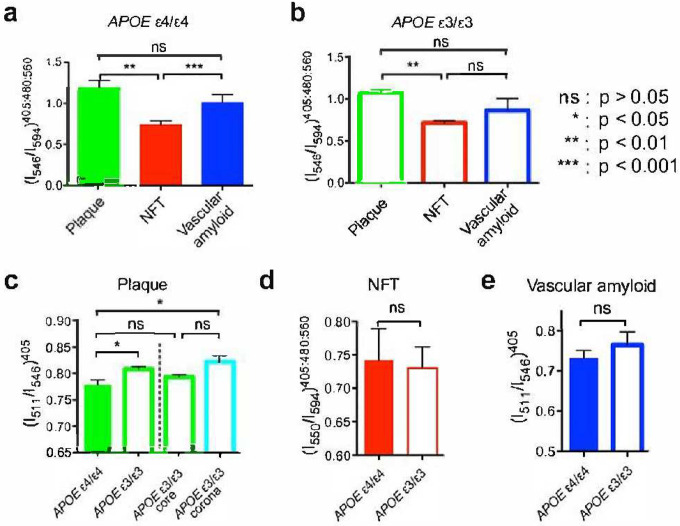
Spectral separation as a function of *APOE* isoform in cohort B. (a) Spectral emission fraction of plaques (green), NFT (red), and vascular amyloid (blue) in AD patients with *APOE* ε4/ε4 genotype. (b) Spectra emission fraction of plaques (green), NFT (red), and vascular amyloid (blue) in AD patients with *APOE* ε3/ε3 genotype. Spectral separation using optimized emission fractions for (c) plaque, (d) NFT, and (e) vascular amyloid in AD patients with *APOE* genotype of ε4/ε4 (filled bars) or respective ε3/ε3 (open bars). The spectral separation of the dense-cored plaques from *APOE* ε3/ε3 AD patients are separated into a core (green) and a corona (cyan) fraction in (c). Error bars indicate SEM.

By employing distinct emission fractions, plaques and NFTs could be distinguished based on the *APOE* isotypes in brain samples from cohort A (Figure 3a–b). In general, plaques and NFTs could also be distinguished by *APOE* isotype in brain samples from cohort B (Figure 5b). The example was that NFTs and vascular amyloid from the *APOE* ε3 homozygous isotype where no significant difference was detected (p = 0.0988, two-tailed paired *t* test). This is likely since vascular amyloid was only found in 3 of the 6 *APOE* ε3 homozygous cases in cohort B.

The emission fraction for plaques could be distinguished depending on the *APOE* isotype (p < 0.01, two-tailed paired *t* test; Figure 3c). A more detailed spectral analysis of the dense-cored plaques for the *APOE* ε3 isotype showed that the spectral differentiation of the *APOE* isotypes was mainly due to a significantly higher value of the emission fraction from the corona surrounding the core of dense-cored plaque (p < 0.01, two-tailed paired *t* test; Figure 1d). The emission fraction from the core was similar to the emission fraction observed for *APOE* ε4 fibrillar plaques (p = 0.2411, two-tailed paired *t* test; Figure 3c). All observations were verified in cohort B (Figure 5c).

As noted, then, the conformation of the dense aggregates (either *APOE* ε3-related spherical cores or *APOE* ε4-related fibrillar cores) were identical between *APOE* genotypes. Instead, the main *APOE* isotype-related differences we observed involved the “macroassembly” structure of the amyloid deposits. Hyman and colleagues have proposed that *APOE* ε4 causes initiation of higher densities of amyloid plaques than does *APOE* ε3 [5]. Conceivably, the irregular and multi-lobar nature of the amyloid plaque type that we observe in *APOE* ε4 homozygotes could be due to confluences of several closely approximated plaque initiation foci that coalesced as more Aβ accretes onto the deposit, causing the plaque foci to expand.

The hyperspectral separation of p-FTAA emission fractions for NFTs according to *APOE* isotype did not reach significance for the pooled data of both cohort A (Figure 3d) or cohort B (Figure 5d).

**Figure 6.**
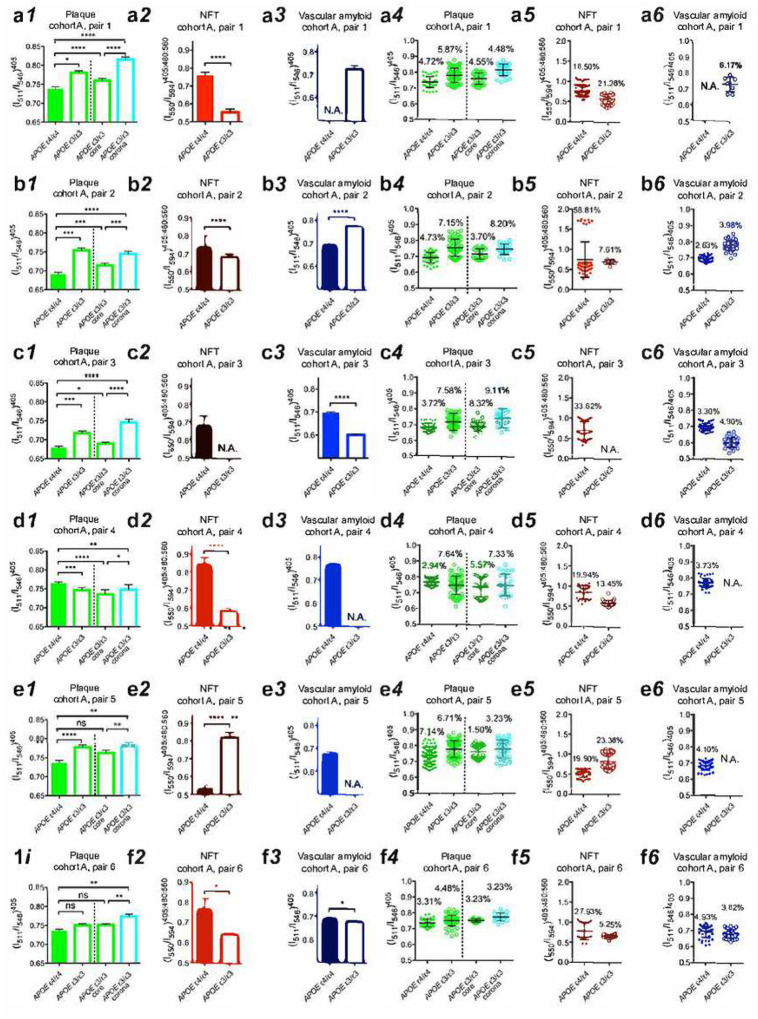
Spectral emission fractions shown for separation and distribution as a function of *APOE* isoform from cohort A of (a) pair 1, (b) pair 2, (c) pair 3, (d) pair 4, (e) pair 5, and (f) pair 6. Spectral separation using optimized emission fractions for (1) plaque, (2) NFT, and (3) vascular amyloid in AD patients with *APOE* genotype of ε4/ε4 (filled bars) or ε3/ε3 (open bars). Spectral distribution optimized emission fractions of (4) plaque, (5) NFT, and (6) vascular amyloid in AD patients with *APOE* genotype of ε4/ε4 (filled circles) or ε3/ε3 (open circles). The spectral emission fraction separation of the dense-cored plaques from *APOE* ε3/ε3 AD patients is separated into one core (green) and one corona (cyan) component in (1) and (4) respectively. Error bars indicate (1–3) SEM and (4–6) SD. Percentage are indicate coefficient of variation in (4–6). Not significant (ns): P > 0.05, *: P < 0.05, **: P < 0.01, ***: P < 0.001, ****: p < 0.0001.

To assess the conformational heterogeneity of pathological aggregates as a function of *APOE* isotype, we also analyzed the spectral variations as the coefficient of variation of the emission fraction within selected patient pairs of cohort A (Table 1) (Figure 6). The larger structural variation of plaques of the *APOE ε3* homozygotes compared to the structural variation of plaques of the *APOE ε4* homozygotes (p < 0.01, two-tailed paired *t* test of coefficient of variation) may reflect the fact that the dense-cored plaques of the *APOE ε3* homozygous samples are composed of a distinct core and a corona while the *APOE ε4* homozygous samples have much larger cores and much narrower corona.

The spectral variation for NFTs in brains of patients homozygous for *APOE* ε4 was unusually high in three cases, and this level of variation was only observed in one sample homozygous for *APOE* ε3 (Figure 6). The source of the spectral variation in NFTs is currently unknown but could be related either to the incorporation of proteins other than tau, or to the incorporation of lipids or ions within the NFTs. The source of the spectral variation in NFTs could also been explained by differential degrees of hyperphosphorylation of tau.

## Discussion

In summary, as expected from amyloid positivity from p-FTAA staining, *APOE* ε4/ε4 cases had a significantly larger load of vascular amyloid than *APOE* ε3/ε3 adding to the notion on impaired clearance of Aβ in *APOE* ε4/ε4 carriers [25]. Furthermore, this investigation based on application of p-FTAA spectral imaging as a surrogate marker for protein structure indicates the existence of *APOE* isotype-dependent differences in the structures of amyloid plaques in human AD postmortem brain.

## Conclusion

These findings support the hypothesis that one mechanism by which the *APOE* ε4 allele modulates the relative risk for AD is by modulating the initiation frequency, initiation density, impair degradation or post-initiation macroassembly of pathological Aβ deposits in brain. These apoE isotype-dependent effects on Aβ-aggregate structures likely influence molecular PET-tracer affinity and efficacy of treatments targeting Aβ-aggregates such as aducanumab and lecanemab and might be relevant to patient stratification and treatment efficacy [26].

## Data Availability

Supporting data will be made available upon request.

## Abbreviations

Aβ: amyloid β peptide
AD: Alzheimer’s disease
*APOE*: apolipoprotein E gene
apoE: apolipoprotein E protein
CAA: cerebral amyloid angiopathy
CDR: clinical dementia rating
LCO: luminescent conjugated oligothiophene
NFTs: neurofibrillary tangles
p-FTAA: (4’,3”’-bis-carboxymethyl-[2,2’;5’,2”;5”,2”’;5”’,2””]quinquethiophene-5,5””-dicarboxylic acid)
